# Evaluation of barley genotypes for drought adaptability: based on stress indices and comprehensive evaluation as criteria

**DOI:** 10.3389/fpls.2024.1436872

**Published:** 2024-08-26

**Authors:** Ruijiao Song, Peichun Shi, Li Xiang, Yu He, Yusheng Dong, Yu Miao, Juncang Qi

**Affiliations:** ^1^ The Key Laboratory of Oasis Eco-agriculture, Xinjiang Production and Construction Group-College of Agriculture, Shihezi University, Shihezi, China; ^2^ Qitai Triticeae Crops Experimental Station, Xinjiang Academy of Agricultural Sciences, Qitai, China

**Keywords:** barley (*Hordeum vulgare* L.), comprehensive evaluation, drought stress, plant adaptability, stress indices

## Abstract

The prevalence of drought events worldwide emphasizes the importance of screening and cultivating drought-adapted crops. In this study, 206 germplasm resources were used as materials, dry weight as target trait, and two genotyping methods as criteria to evaluate drought adaptability at the seedling establishment stage. The results showed a significant decrease in average dry weight of the tested germplasm resources (from 746.90 mg to 285.40 mg) and rich variation in the responses of dry weight among each genotype to drought (CV=61.14%). In traditional evaluation method, drought resistance coefficient (DC), geometric mean productivity index (GMP), mean productivity index (MP), stress susceptibility index (SSI), stress tolerance index (STI), and tolerance index (TOL) also exhibited diversity in tested genotypes (CV>30%). However, these indices showed varying degrees of explanation for dry weight under stress and non-stress environments and failed to differentiate drought adaptability among genotypes clearly. In new evaluation method, four stress indices were developed to quantify barley seedling production and stability capacities. Compared to traditional stress indices, the stress production index (SI) explained dry weight more comprehensively under stress conditions (*R^2 ^=* 0.98), while the ideal production index (II) explained dry weight better under non-stress conditions (*R^2 ^=* 0.89). Furthermore, the potential index (PI) and elasticity index (EI) eliminated disparities in traditional stress indices and comprehensively clarified the contribution of elasticity and potential to production capacity under drought stress. Ultimately, through grading evaluation and cluster analysis, the tested germplasm resources were effectively categorized, and 11 genotypes were identified as suitable for cultivation in arid areas. Overall, the comprehensive evaluation method based on the newly developed stress indices surpasses the traditional method in screening drought adaptability of crops and serves as a vital tool for identifying high-stability and high-production capacities genotypes in various environments, which is expected to provide practical guidance for barley planting and breeding in arid areas.

## 1 Introduction

Barley (*Hordeum vulgare* L.), one of the most widely and oldest domesticated crops, has been cultivated for over 10,000 years (Haas et al., 2019). It possesses significant ecological and nutritional value and plays crucial roles in providing food for human beings, raw materials for malt and beer industries, and feed for animal husbandry and aquaculture (Elakhdar et al., 2022; Newton et al., 2011). In 2021, global barley production exceeded 145 million tons, ranked the fourth-largest cereal crop after maize, rice, and wheat, according to the Food and Agriculture Organization of the United Nations (FAO, 2023).

With the intensification of climate change and water scarcity, drought has become one of the strongest stress factors restricting crop production (Leng and Hall, 2019). Although barley is considered to have great adaptability to abiotic stress, it remains susceptible to drought during the seedling establishment stage (Sallam et al., 2019). Unfortunately, most barley-growing areas are facing the challenge of insufficient water supply. Once drought occurs at the early growth stage of crop, weak seedlings are often formed, even unable to emerge, thus hindering the formation of subsequent yields (Wehner et al., 2016). Furthermore, plants also have to face other challenges even in rainfall-abundant areas, such as extreme temperatures (high or low) and exacerbated salinization issues, which can disrupt the water absorption capacity of seedlings, resulting in physiological drought (Cammarano et al., 2019; Liu et al., 2022b; Perri et al., 2020). Currently, many strategies have been implemented to address these challenges (Elakhdar et al., 2022; Lankford et al., 2023). Among them, evaluating and cultivating existing germplasm resources is a promising approach to developing drought adaptation potential (Quevedo et al., 2022).

Genotypes that demonstrate high productivity in both normal and stress environments are considered excellent in germplasm resource screening and improvement programs (Raman et al., 2012). Toward this goal, breeders widely accepted a classical assumption in their selection process that genotypes with high production under non-stress environments also exhibit good performance under stress environments (Blum, 2005). However, this selection method fails to consider adaptation to stressful environments and lacks the concept of stability. Various stress indices have been used to evaluate the environmental adaptability of crops in recent years, including drought resistance coefficient (DC), geometric mean productivity index (GMP), mean productivity index (MP), stress susceptibility index (SSI), stress tolerance index (STI), tolerance index (TOL), and so on (Ayed et al., 2021; Li et al., 2023). Among them, GMP, MP, and TOL had high correlation and could be used to identify the relative salt tolerance of barley varieties (Jamshidi and Javanmard, 2018). In comparison, STI and SSI could more effectively screen the genotypes that are high productivity or less affected by stress (Lan et al., 2022; Sánchez-Reinoso et al., 2020). Additionally, DC, as a common stress index was widely used to directly screen or comprehensively evaluate drought-resistant germplasm resources in various plants (Chen et al., 2014; Zou et al., 2020). These indices account for the relationship between traits in non-stress and stress environments. However, none of them are ideal indices to measure crop stability and fail to effectively classify the four types of production performance: A) performing well in both non-stress and stress environments; B) performing well only in non-stress environments; C) performing well only in stress environments; and D) performing poorly in both non-stress and stress environments (Fernandez, 1992). Up to now, there is no accurate screening index that can be recommended for genotype selection in high productivity and high stability breeding programs in both stressed and non-stressed environments. Based on this, some researchers believed that using combinations of existing stress indices could provide more effective criteria for evaluating stress adaptation (Aberkane et al., 2021; Sabouri et al., 2022). However, how to properly integrate stress indices remains a subject of limited understanding and requires further exploration.

In this study, we employed a comprehensive dataset of seedling biomass from 206 barley germplasms to calculate the values of DC, GMP, MP, SSI, STI, and TOL for each genotype. Subsequently, these indices were combined using correlation analysis, normalization treatment, and principal component analysis. Finally, the drought adaptability of all tested germplasm resources was then comprehensively evaluated through grading evaluation and cluster analysis. The primary aims of this study are: 1) to analyze the feasibility of employing six traditional stress indices as evaluation criteria for drought adaptability in barley; 2) to establish an intuitive method that can comprehensively assess drought adaptability considering both productivity and stability performances while being applicable for high-throughput screening of barley germplasms at early growth stage; and 3) to identify genotypes with high productivity and high stability across various environments and recommend them for cultivation in arid areas or as valuable resources for breeding programs.

## 2 Materials and methods

### 2.1 Experimental plant material

206 barley germplasms were used in this study (**Supplementary Table 1**). All materials were harvested from the Experimental Station of Agricultural College of Shihezi University in 2021.

### 2.2 Experimental design and plant culture

Uniform-sized and intact seeds were selected and then soaked in 75% alcohol for 30 s and 10% sodium hypochlorite for 10 min. Subsequently, the seeds were thoroughly rinsed with running water to remove residual disinfectant and then immersed in distilled water under dark conditions for 24 h. After whiteness, the seeds were carefully placed in transparent boxes (10×15 cm) with two layers of filter paper. Each box was filled with 50 seeds, and 40 mL of distilled water or a 20% polyethylene glycol-6000 (w/v) solution was added. The above boxes were placed in artificial climate incubators (Prandt, Ningbo, China) cultured for 7 d under 25°C conditions with 12 h/12 h light/dark cycle at light intensity of 400 µmol·m^2^·s^-1^ (Song et al., 2023). Each treatment was replicated three times.

### 2.3 Determination of dry weight

Upon reaching the harvest period, the transparent boxes were removed from the artificial climate incubators. The seedlings were rinsed with distilled water to remove residual culture solution from their surfaces and then gently wiped using filter paper. The total seedlings fresh weight of each box was measured using a precision balance with a sensitivity of 1/10,000 (Sartorius, Beijing, China). Following this, the materials were placed in kraft paper bags and put into the drying oven (Ever bright, Beijing, China) at 105°C for 30 min. The temperature was then adjusted to 80°C for further drying until a constant weight was obtained. Finally, the plants were removed from the drying oven, weighed, and the average dry weight of each treatment was calculated (Jia et al., 2020).

### 2.4 Calculation of stress indices

Stress indices were calculated from the dry weight data using the methods described by Sánchez-Reinoso et al. (2020) and Zou et al. (2020). The modified equations are described below:


(1) $$DC=\frac{T}{C}$$

(2) $$GMP=\sqrt{T\times C}$$

(3) $$MP=\frac{T+C}{2}$$

(4) $$SSI=\frac{1-\left(\frac{T}{C}\right)}{1-\left(\frac{\bar{T}}{\bar{C}}\right)}$$

(5) $$STI=\frac{C\times T}{{\left(\bar{C}\right)}^{2}}$$

(6) TOL=C−T

C is the average dry weight of each genotype under control conditions; T is the average dry weight of each genotype under drought conditions; $\bar{\text{C}}$ is the average dry weight of 206 genotypes under control conditions; $\bar{\text{T}}$ is the average dry weight of 206 genotypes under drought conditions.

### 2.5 Normalization of stress indices

The six stress indices were feature scaled using two normalization equations, denoted as follows:


(7) $$x_{n}=\frac{\left(x-x_{min}\right)}{\left(x_{max}-x_{min}\right)}$$

(8) $$y_{n}=\frac{\left(y_{max}-y\right)}{\left(y_{max}-y_{min}\right)}$$

DC, GMP, MP, and STI used Equation 7 (min-max scaling method). $x_{n}$ is the normalized index value of each genotype; x is the original index value of each genotype; $x_{max}$ is the maximum original index value of 206 genotypes; $x_{min}$ is the minimum original index value of 206 genotypes.

SI and TOL used Equation 8 (negative min-max scaling method). $y_{n}$ is the normalized index value of each genotype; y is the original index value of each genotype; $y_{max}$ is the maximum original index value of 206 genotypes; $y_{min}$ is the minimum original index value of 206 genotypes.

### 2.6 Data analysis

Data were recorded and calculated in Microsoft Excel 2021 (Microsoft, Redmond, USA). Group comparisons were tested by paired sample t-test using the IBM SPSS Statistics 19 (SPSS, Chicago, USA). Graphs were processed with Microsoft Excel 2021 (Microsoft, Redmond, USA) and Microsoft Office PowerPoint 2021 (Microsoft, Redmond, USA). Website tools ChiPlot (https://www.chiplot.online/) and Hiplot (https://hiplot.com.cn/) were utilized when necessary.

## 3 Results

### 3.1 Response of seedling dry weight to drought stress

**Figure 1:**
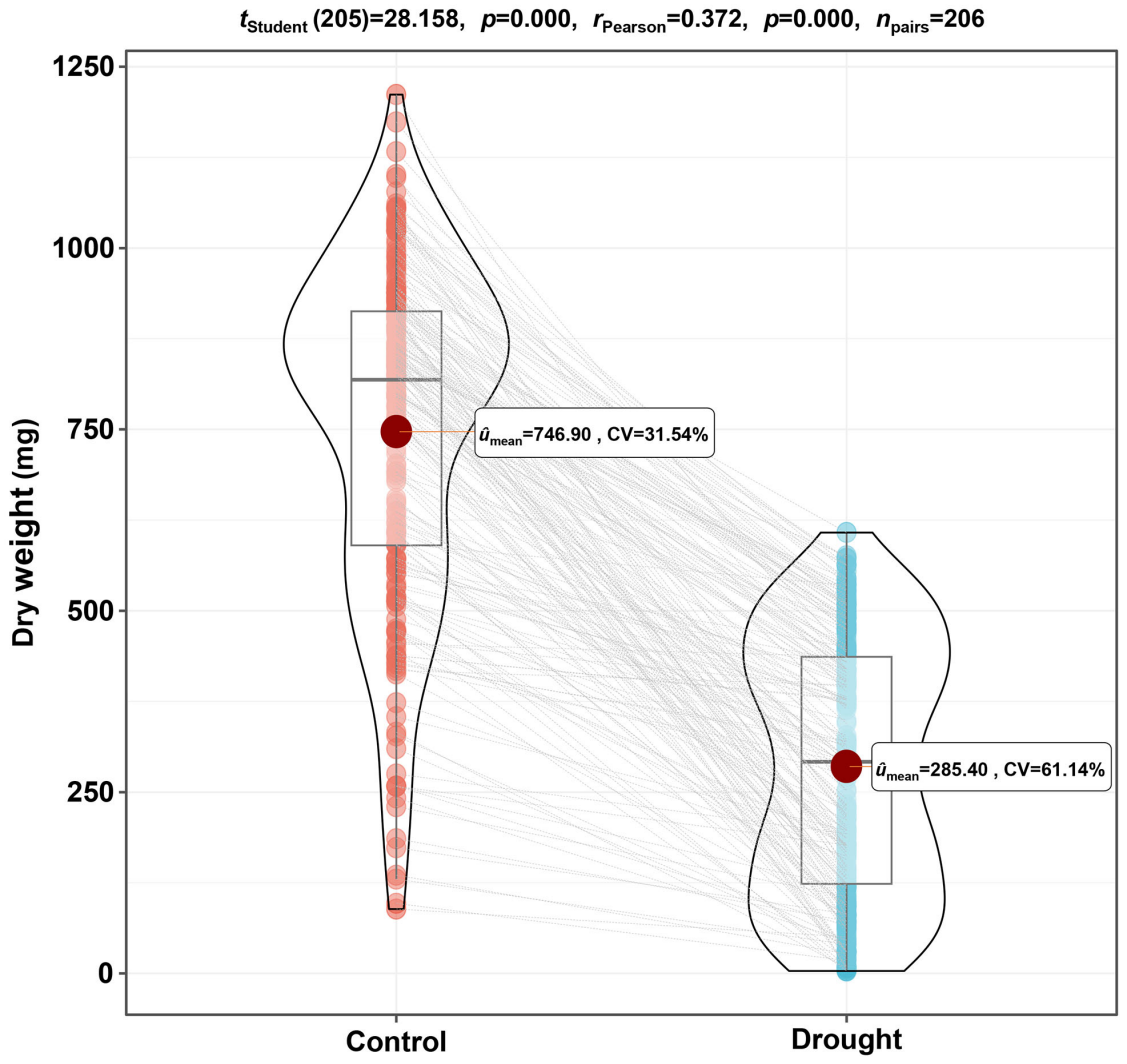
The differences in dry weight of 206 barley germplasms under control and drought conditions. Barley seeds of 206 genotypes were pre-germinated for 1 d and then cultured in water or 20% polyethylene glycol solution for 7 d, and the dry weight of seedlings was measured after harvesting. The paired sample t-test was performed based on the average dry weight of three replicates for each germplasm. CV, coefficient of variation.

Based on the dry weight, which is an essential trait during the seedling establishment stage, a drought response analysis was conducted on 206 barley germplasms. As shown in **Figure 1**, the average dry weight under the drought condition exhibited a decrease, from 746.90 mg to 285.40 mg, with a reduction of 61.79% compared to the control. The inhibitory effect on dry weight for drought is highly significant (*p*<0.001), indicating that 20% polyethylene glycol is suitable as a screening condition for drought-adapted germplasms in this experiment. What is more, the coefficient of variation (CV) among different germplasms under control and drought conditions were 31.54% and 61.14%, respectively, demonstrating extensive variation among the 206 germplasms under stress and non-stress environments, and the populations used in the experiment are representative.

### 3.2 Evaluation of seedling dry weight by different stress indices

**Figure 2:**
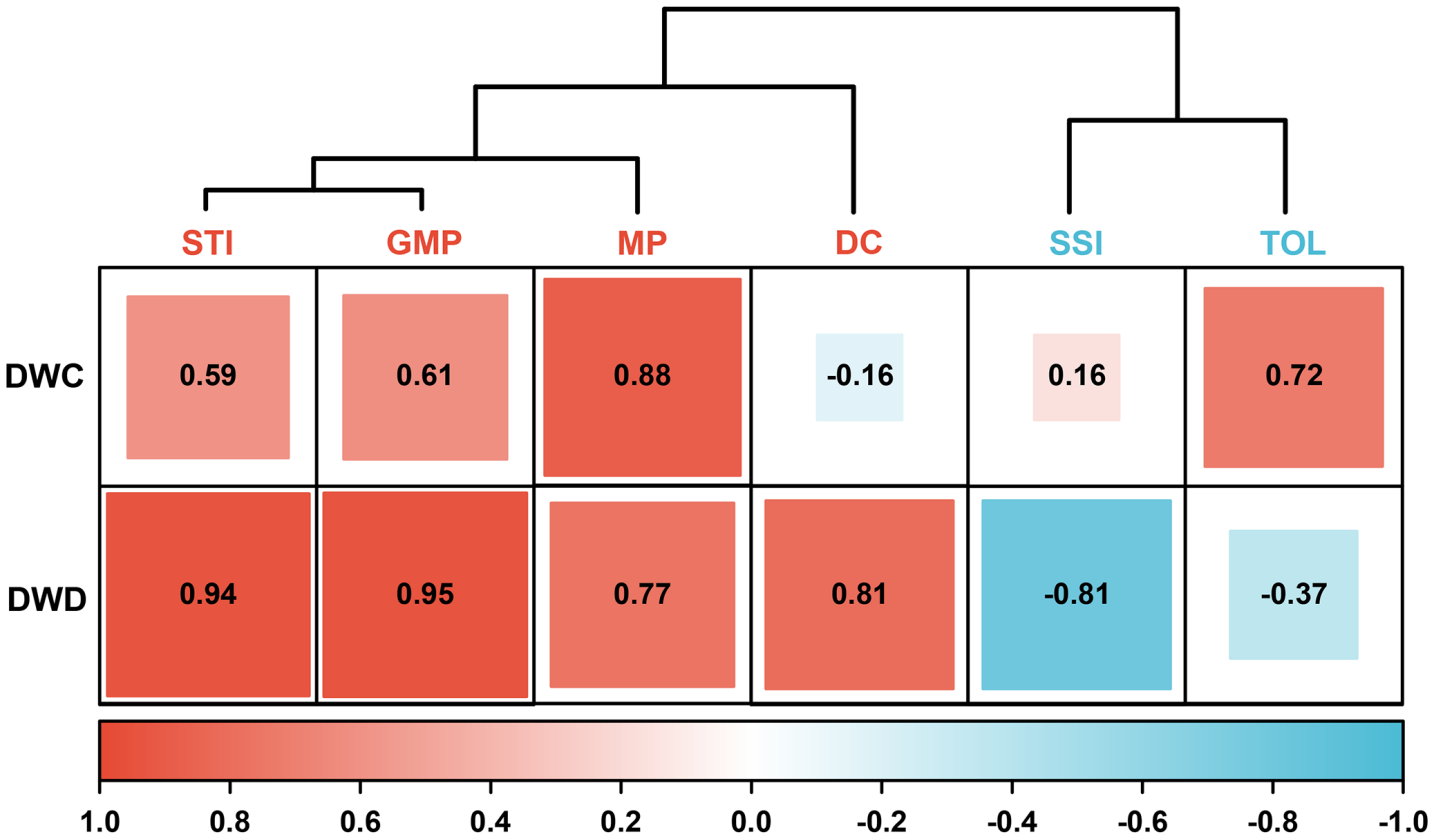
The correlation cluster analysis with six stress indices and dry weight of barley seedling. DC, drought resistance coefficient; GMP, geometric mean productivity index; MP, mean productivity index; SSI, stress susceptibility index; STI, stress tolerance index; TOL, tolerance index; DWC, dry weight under control conditions; DWD, dry weight under drought conditions.

**Figure 3:**
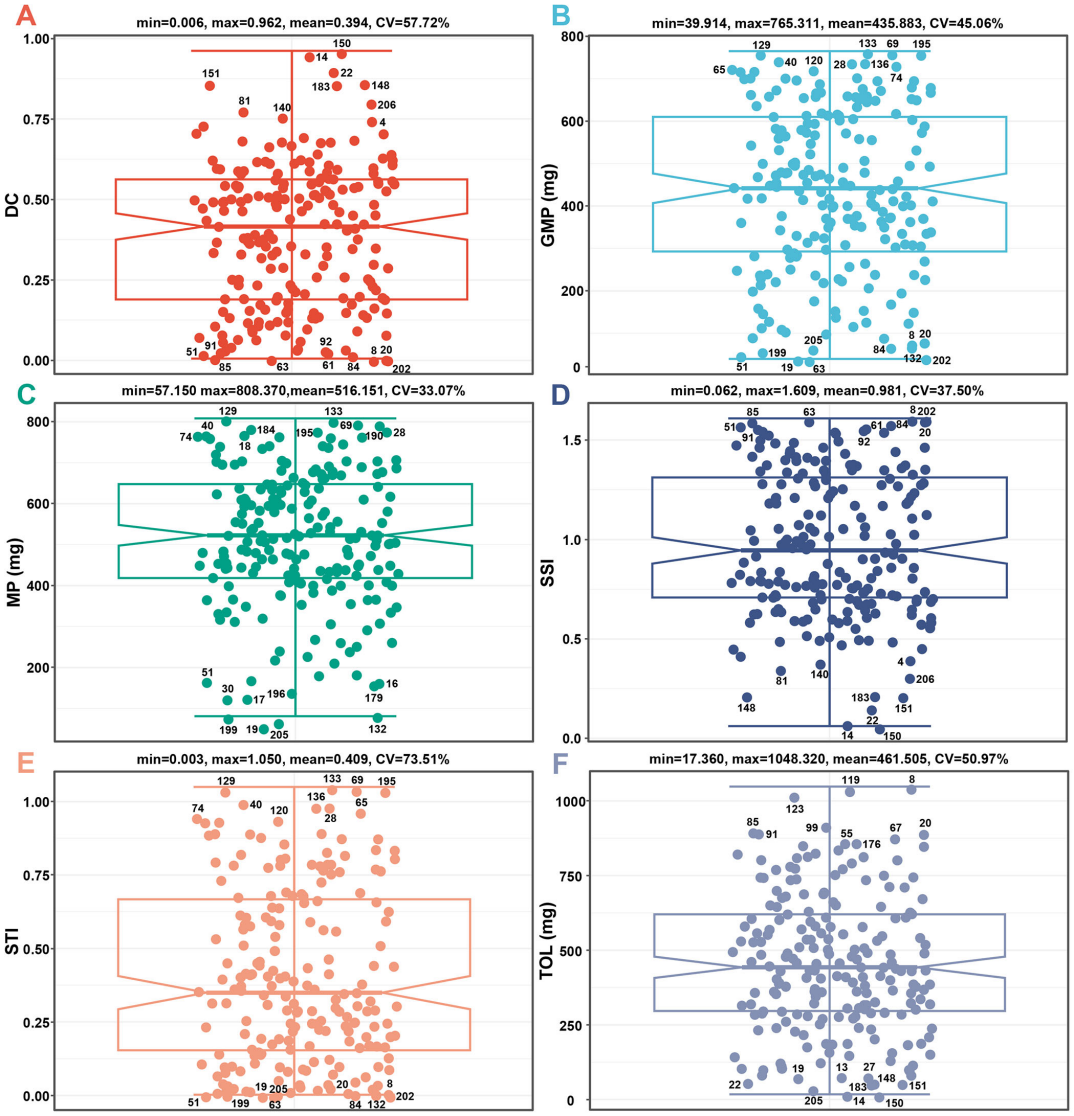
The differences in DC **(A)**, GMP **(B)**, MP **(C)**, SSI **(D)**, STI **(E)**, and TOL **(F)** values of 206 barley seedlings. CV, coefficient of variation; DC, drought resistance coefficient; GMP, geometric mean productivity index; MP, mean productivity index; SSI, stress susceptibility index; STI, stress tolerance index; TOL, tolerance index.

DC, GMP, MP, SSI, STI, and TOL are crucial indices for assessing the drought adaptation level of crops. As shown in **Figure 2**, these indices are divided into two types. DC, GMP, MP, and STI are positively correlated with dry weight, and higher values indicate a higher adaptability to drought. Conversely, SSI and TOL are negatively correlated with dry weight, and higher values denote lower adaptability to drought. The CV for all stress indices falls within the range of 33.07% to 73.51%, indicating that the above indices apply to the evaluation of test samples (**Figure 3**).

DC is the ratio of dry weight under drought and control conditions. The DC values of the tested materials range from 0.006 to 0.962, with a mean of 0.394. Among them, genotypes 8, 202, 63, 20, 85, 84, 51, 61, 91, and 92 exhibit the lowest DC values, while genotypes 150, 14, 22, 151, 148, 183, 206, 81, 140, and 4 exhibit the highest DC values (**Figure 3A**).

GMP is the square root of the product of dry weight for seedlings under drought and control conditions. The GMP values of the tested materials range from 39.914 to 765.311, with a mean of 435.883. Among them, genotypes 63, 19, 202, 51, 199, 205, 84, 132, 8, and 20 had the lowest GMP values, while genotypes 133, 69, 129, 195, 40, 136, 28, 65, 74, and 120 had the highest GMP values (**Figure 3B**).

MP is the average dry weight of seedlings under drought and control conditions. The MP values of the tested materials range from 57.150 to 808.370, with a mean of 516.151. Among them, genotypes 19, 205, 199, 132, 30, 17, 196, 179, 16, and 51 had the lowest MP values, while genotypes 129, 133, 69, 190, 184, 195, 28, 40, 18, and 74 had the highest MP values (**Figure 3C**).

SSI is the stress sensitivity of each genotype within the entire sample. The SSI values of the tested materials range from 0.062 to 1.609, with a mean of 0.981. Among them, genotypes 150, 14, 22, 151, 148, 183, 206, 81, 140, and 4 had the lowest SSI values, while genotypes 8, 202, 63, 20, 85, 84, 51, 61, 91, and 92 had the highest SSI values (**Figure 3D**).

STI is the stress tolerance of each genotype within the entire sample. The STI values of the tested materials range from 0.003 to 1.050, with a mean of 0.409. Among them, genotypes 63, 19, 202, 51, 199, 205, 84, 132, 8, and 20 had the lowest STI values, while genotypes 133, 69, 129, 195, 40, 136, 28, 65, 74, and 120 had the highest STI values (**Figure 3E**).

TOL is the variation of dry weight under control and drought conditions. The TOL values of the tested materials range from 17.360 to 1048.320, with a mean of 461.505. Among them, genotypes 150, 14, 205, 22, 151, 183, 148, 19, 13, and 27 had the lowest TOL values, while genotypes 8, 119, 123, 99, 85, 91, 20, 67, 55, and 176 had the highest TOL values (**Figure 3F**).

Based on the screening results of all positive stress indices (DC, GMP, MP, and STI), the genotypes with the highest drought adaptability are 28, 40, 133, 69, 195, 74, 129, 65, 120, and 136, while the genotypes with the lowest drought adaptability are 51, 8, 202, 63, 20, 84, 19, 199, 205, and 132. Based on the screening results of all negative stress indices (SSI and TOL), the genotypes with the highest drought adaptability are 150, 14, 22, 151, 148, and 183, while the genotypes with the lowest drought adaptability are 8, 20, 85, and 91 (**Figure 3**). Overall, there is no overlap in genotype for high drought adaptability between the two types of stress indices.

### 3.3 Establishment of a comprehensive evaluation method

#### 3.3.1 Establishment and grading evaluation of stability indices

**Figure 4:**
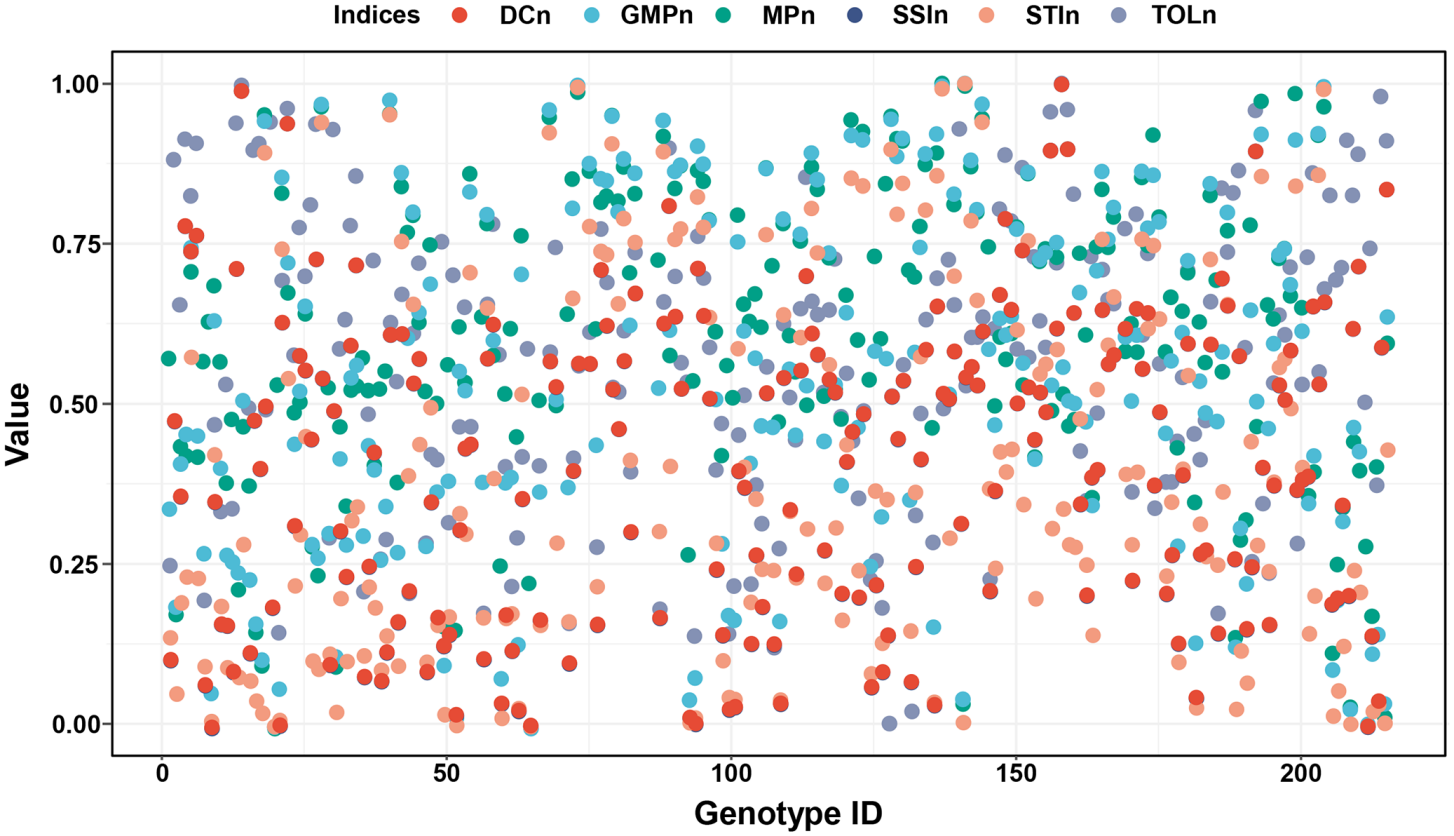
The normalized values of DC, GMP, MP, SSI, STI, and TOL. Positive stress indices (DC, GMP, MP, and STI) were used the min-max scaling method to feature scale and obtained DCn, GMPn, MPn, and STIn. Negative stress indices (SSI, TOL) were normalized using the negative min-max scaling method to feature scale and obtained SSIn and TOLn. DC, drought resistance coefficient; GMP, geometric mean productivity index; MP, mean productivity index; SSI, stress susceptibility index; STI, stress tolerance index; TOL, tolerance index.

To eliminate the differences in dimension, order of magnitude, and action direction, the normalized method was used to feature scale the values of six stress indices. As shown in **Figure 4**, all normalized indices values are from 0 to 1 and have a consistent direction of action, where higher values indicate greater drought adaptability of the corresponding genotypes.

**Figure 5:**
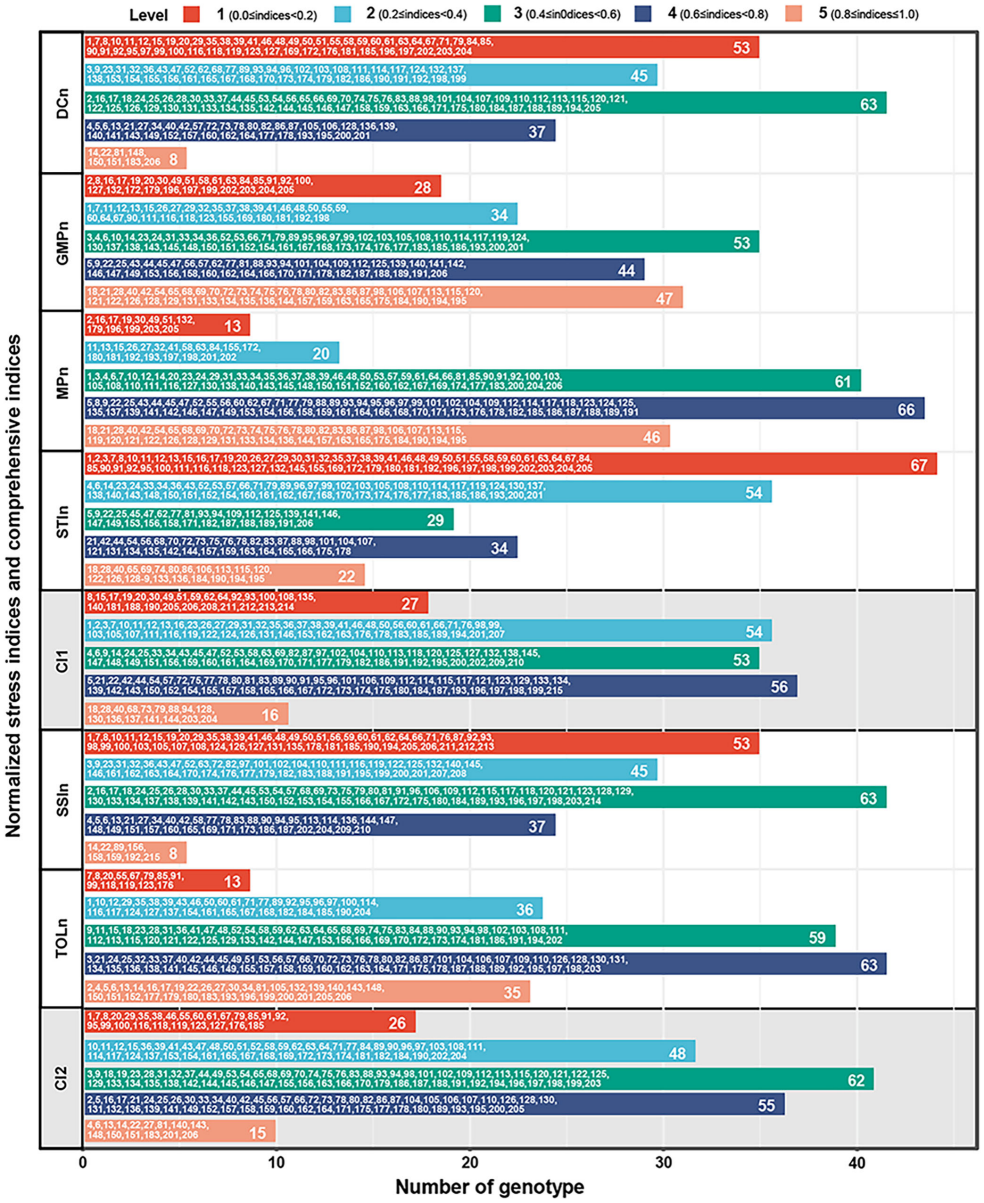
The grading evaluation of CI1, CI2, DCn, GMPn, MPn, SSIn, STIn, and TOLn. CI1, comprehensive stress index 1, is the mean of DCn, GMPn, MPn, and STIn. CI2, comprehensive stress index 2, is the mean of SSIn and TOLn. All indices were divided into five levels according to the full range, in which 0≤indices<0.2 is level 1, 0.2≤indices<0.4 is level 2, 0.4≤indices<0.6 is level 3, 0.6≤indices<0.8 is level 4, and 0.8≤indices≤1.0 is level 5. DCn, normalized drought resistance coefficient; GMPn, normalized geometric mean productivity index; MPn, normalized mean productivity index; SSIn, normalized stress susceptibility index; STIn, normalized stress tolerance index; TOLn, normalized tolerance index.

To accurately evaluate the differences in drought adaptability among 206 germplasms, the comprehensive indices were developed and graded based on the values of two types of normalized stress indices. The results demonstrated that grading evaluation provided a more intuitive reflection of the drought adaptability of each genotype compared to simply ranking the values of the stress indices. As shown in **Figure 5**, there are variations in the number of genotypes across different levels of each stress index. For the four positive stress indices, the number of genotypes in GMPn and MPn is the fewest at level 1 (28 and 13, respectively) while the largest at level 3 (53) and level 4 (66). Furthermore, the number of genotypes in DCn and STIn is the fewest at level 5 (8 and 22, respectively), while the largest are at level 3 (63) and level 1 (67). In comparison, the comprehensive stress index 1 (CI1) integrates the information from the above four indices, indicating a distribution trend with fewer genotypes on both sides and a higher one in the middle. Among them, the number of genotypes at level 1 and level 5 is 27 and 16, respectively, while at levels 2, 3, and 4 exceeded 50. For the two negative stress indices, the number of genotypes in SSIn exhibited the fewest at level 5 (8) and the largest at level 3 (63). In contrast, the number of genotypes in TOLn exhibited the fewest at level 1 (13) and the largest at level 4 (63). In comparison, the comprehensive stress index 2 (CI2) integrates the information from the above two indices, showing a distribution pattern with more in the middle and fewer on the sides, where level 1 and 5 genotypes are the least (26 and 15, respectively), and level 3 genotypes are the most (62).

**Figure 6:**
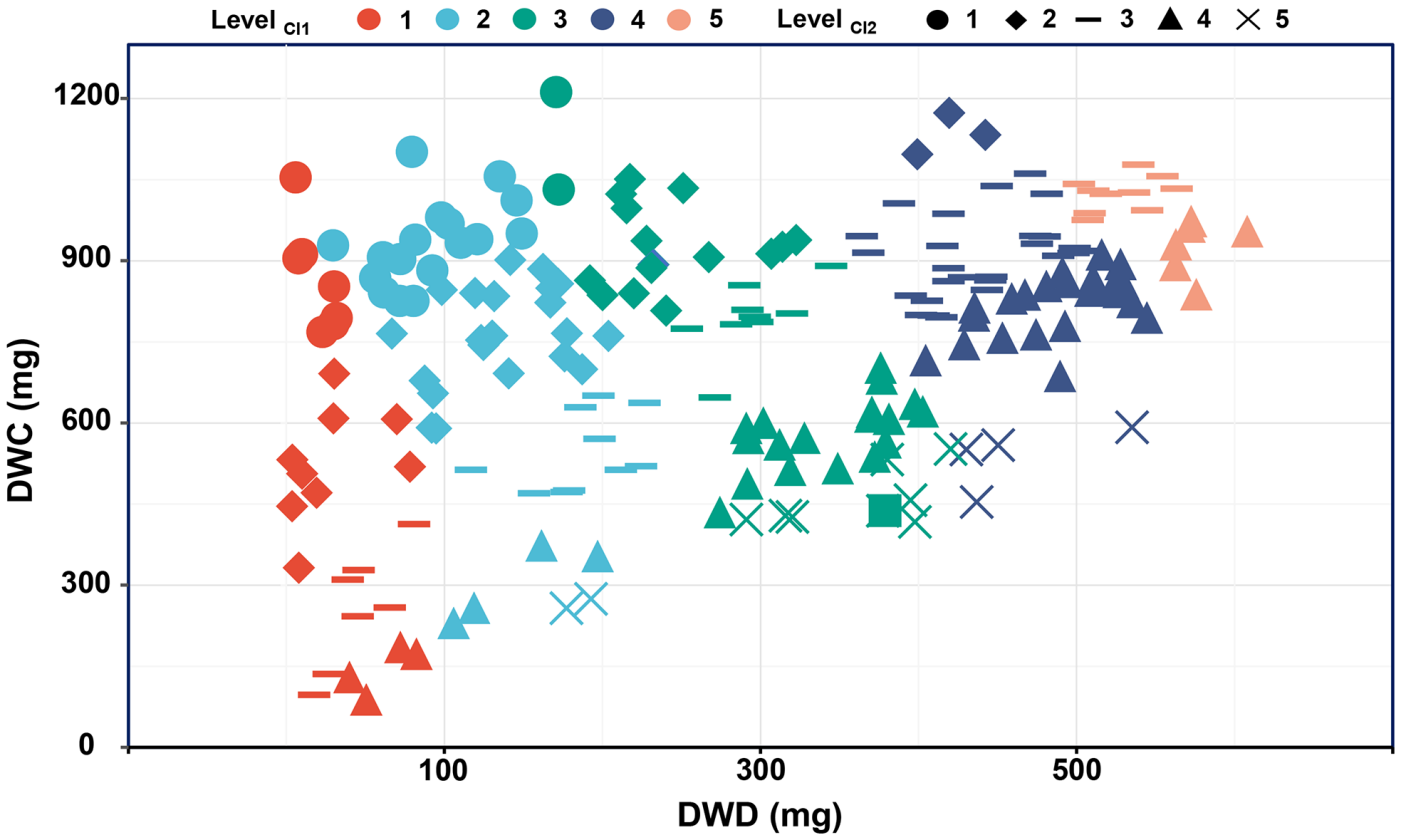
The relationship between dry weight in different environments and CI1 and CI2 grading evaluation. CI1, comprehensive stress index 1; CI2, comprehensive stress index 2; DWC, dry weight under control conditions; DWD, dry weight under drought conditions.

Despite CI1 and CI2 demonstrating similar distribution patterns, that is, a limited number of genotypes show extreme drought stability (levels 1 and 5), and the majority display general drought stability (levels 2, 3, 4). However, there is still no overlap in the high-stability genotypes (level 5) selected by CI1 and CI2. Furthermore, additional analysis reveals that CI1 focuses on identifying high productivity and tolerance, while CI2 emphasizes the differentiation between tolerance and sensitivity. Therefore, CI1 is named the potential index (PI), and CI2 is the elasticity index (EI) in this study (**Figure 6**).

#### 3.3.2 Establishment and grading evaluation of productivity indices

**Figure 7:**
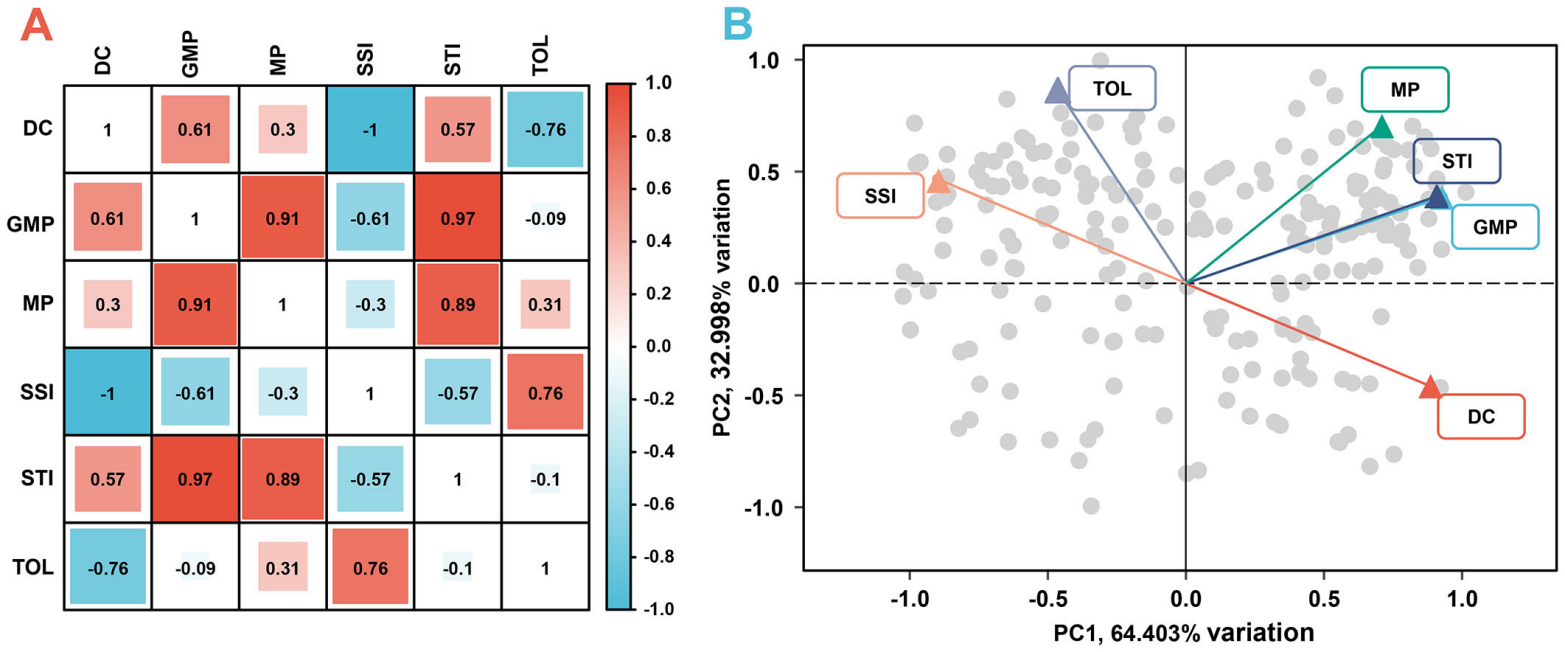
The correlation analysis **(A)** and principal component analysis **(B)** of six stress indices. DC, drought resistance coefficient; GMP, geometric mean productivity index; MP, mean productivity index; SSI, stress susceptibility index; STI, stress tolerance index; TOL, tolerance index; PC1, principal component 1; PC2, principal component 2.

**Figure 8:**
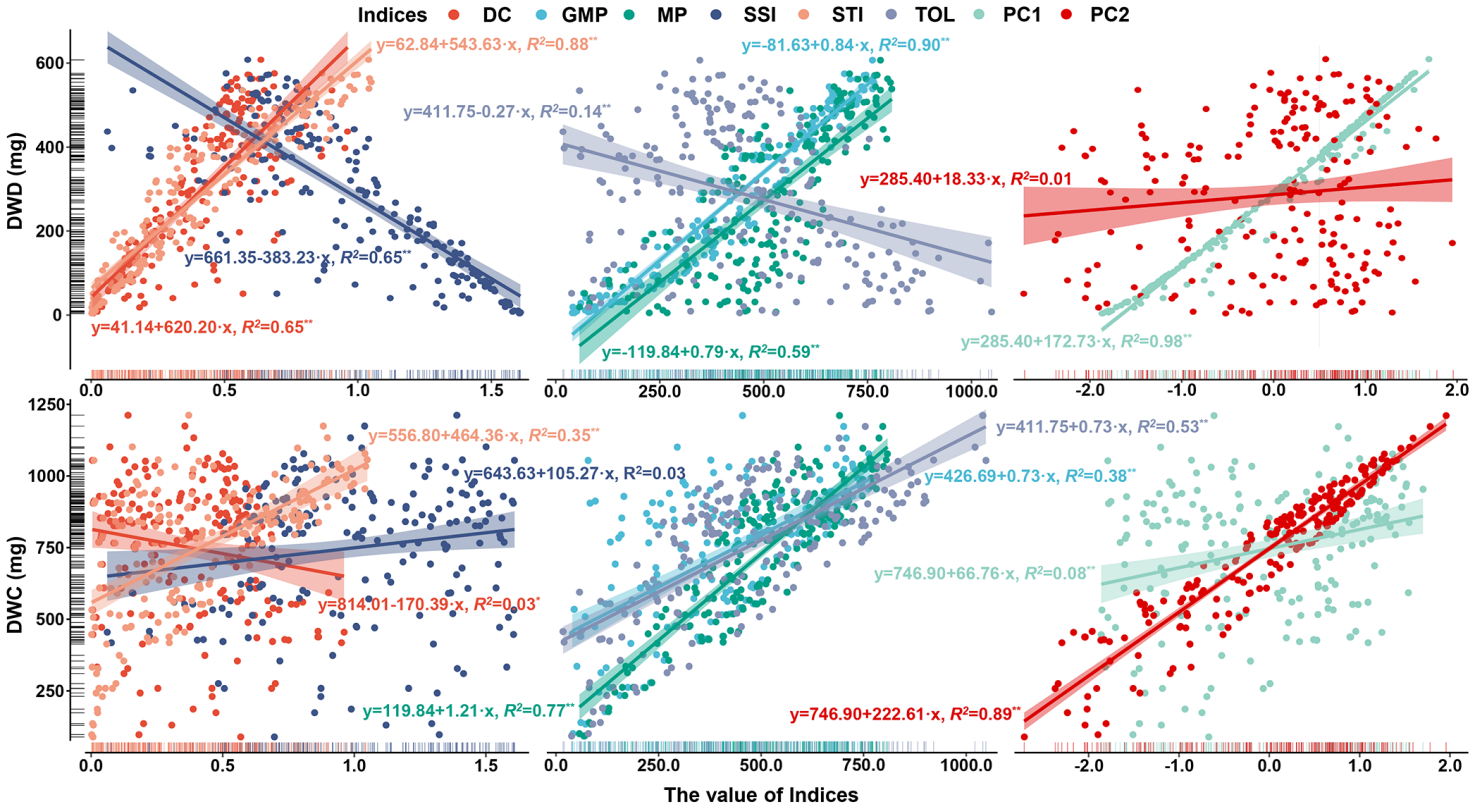
The regression analysis between DC, GMP, MP, SSI, STI, TOL, PC1, PC2, and dry weight of barley seedlings under control and drought conditions. *represents significant at 0.05 level. **represents significant at 0.01 level. DC, drought resistance coefficient; GMP, geometric mean productivity index; MP, mean productivity index; SSI, stress susceptibility index; STI, stress tolerance index; TOL, tolerance index; PC1, principal component 1; PC2, principal component 2; DWC, dry weight under control conditions; DWD, dry weight under drought conditions.

Productivity under non-stress and stress environments is the main criterion for evaluating drought adaptability. To establish the new indices that can better characterize the dry weight of genotypes in various environments, dimension reductions of DC, MP, GMP, SSI, STI, and TOL were performed. **Figure 7A** shows a strong correlation among the above six stress indices, satisfying the requirement for principal component analysis. Accordingly, the principal component analysis result reveals that the extraction of two principal components contributes most to the explained variance, where the contribution rate of principal component 1 (PC1) is 64.403% and that of principal component 2 (PC2) is 32.998%. The cumulative contribution rate of 97.401% indicates that these two principal components can contain almost all the variation information of stress indices (**Figure 7B**). **Figure 8** illustrates the varying degrees of characterization of crop biomass by DC, GMP, MP, SSI, STI, and TOL in different conditions. The determination coefficients (*R^2^*) of the linear regression between them and dry weight under control conditions are 0.03, 0.38, 0.77, 0.03, 0.35, and 0.53, respectively, much lower than those of PC2 (0.89). Under drought conditions are 0.65, 0.90, 0.59, 0.65, 0.88, and 0.14, respectively, much lower than those of PC1 (0.98).

**Figure 9:**
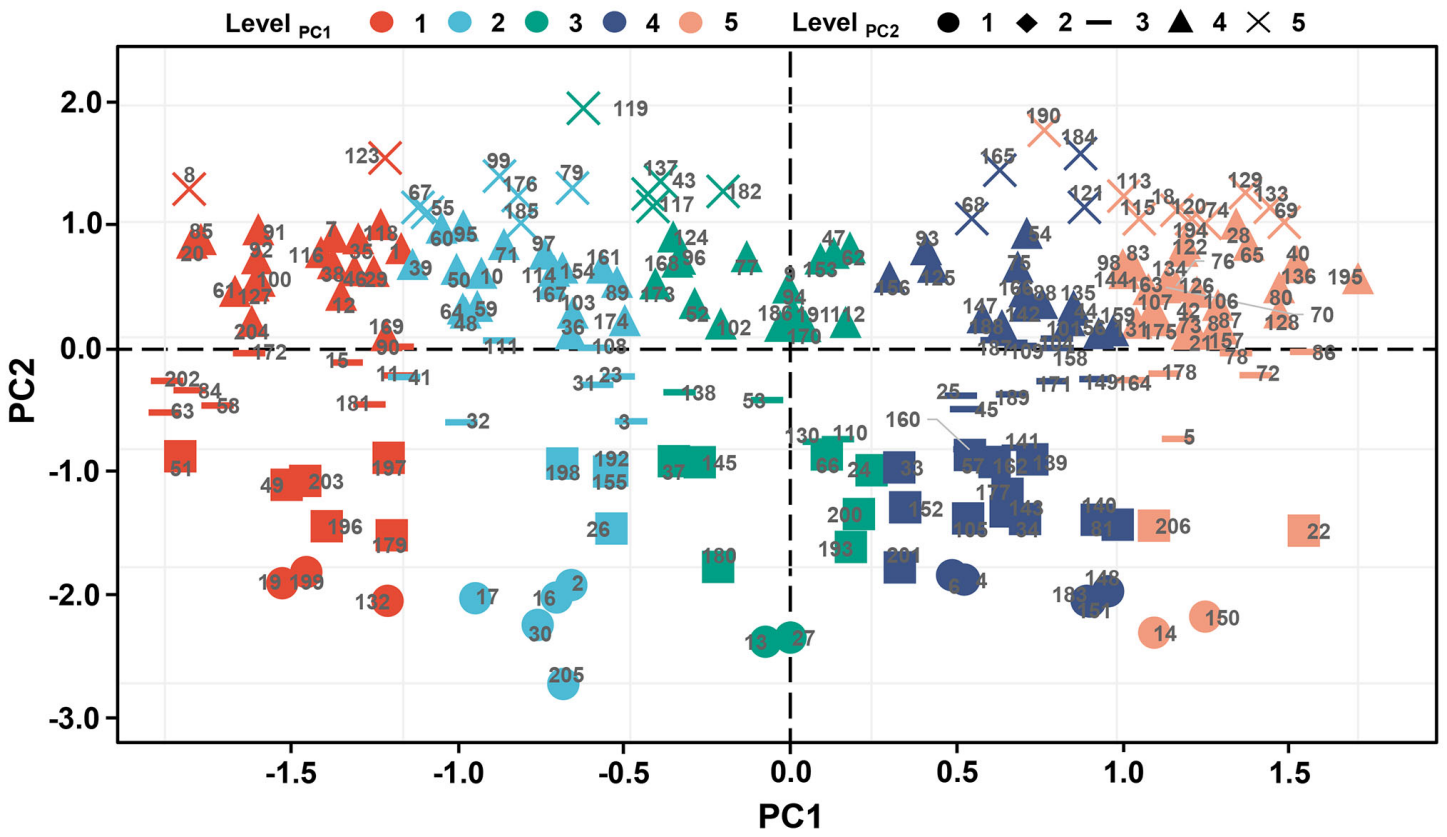
The grading evaluation of PC1 and PC2. All indices were divided into five levels according to the full range, in which the first 20% is level 1, 20%-40% is level 2, 40%-60% is level 3, 60-80% is level 4, and the last 20% is level 5. PC1, principal component 1; PC2, principal component 2.

Given the meaning of the two principal components, PC1 is defined as the stress production index (SI) and PC2 as the ideal production index (II). Moreover, SI and II are divided into five levels equally based on the full range, with the highest-level genotypes being 18, 69, 74, 113, 115, 120, 129, 133, and 190 and the lowest-level genotypes being 19, 132, and 199 (**Figure 9**).

#### 3.3.3 Evaluation of drought adaptability of barley seedlings

**Figure 10:**
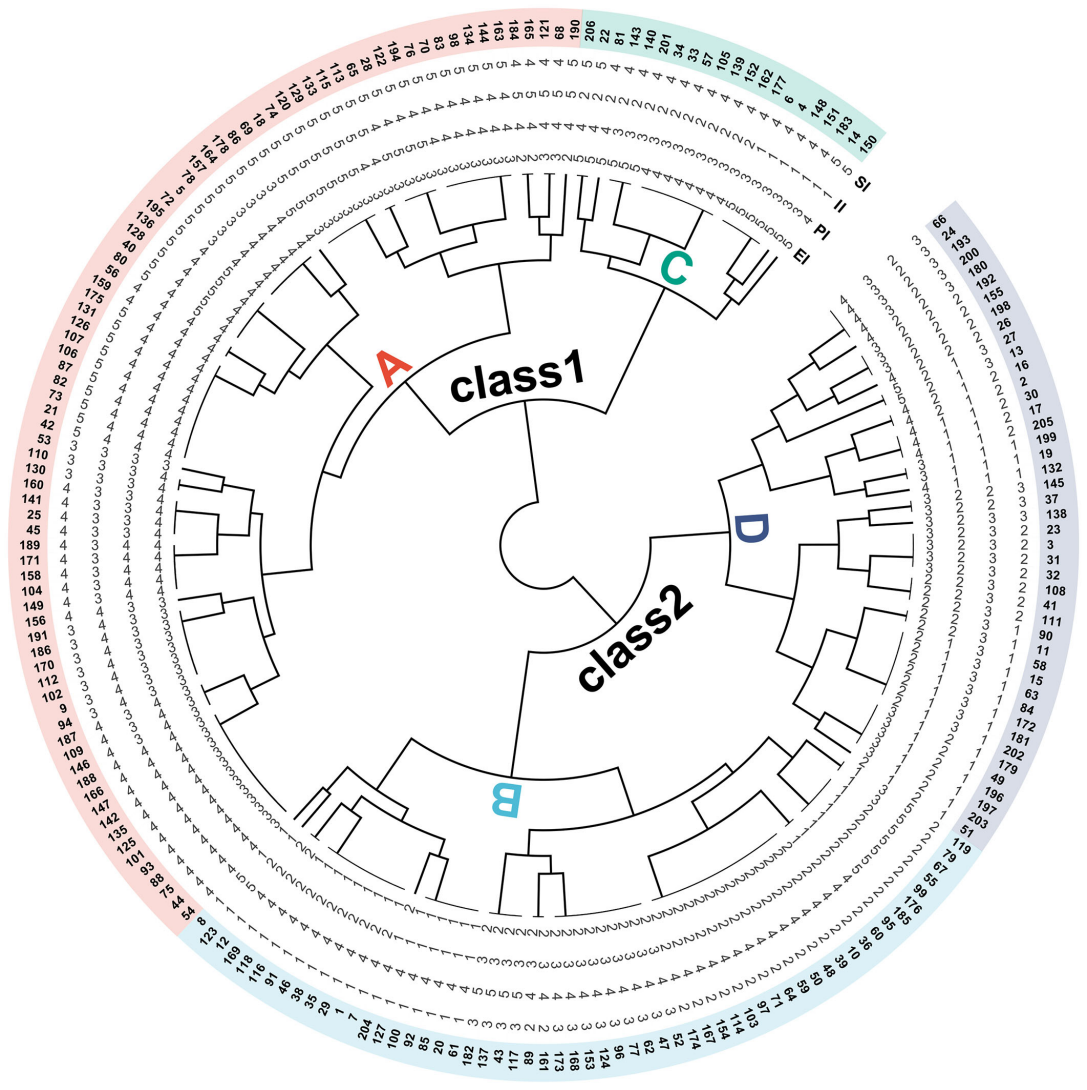
The cluster analysis of the grading evaluation of SI, II, PI, and EI. Different numerical levels correspond to differences in the ability represented by SI, II, PI, and EI: extremely high (level 5), high (level 4), medium (level 3), low (level 2), and extremely low (level 1). Different capital letters correspond to four types of drought adaptation performance: type A (performing well in both non-stress and stress environments), type B (performing well only in non-stress environments), type C (performing well only in stress environments), type D (performing poorly in both non-stress and stress environments). SI, stress production index; II, ideal production index; PI, potential index; EI, elasticity index.

To visually evaluate the drought adaptability of barley seedlings across different genotypes, the strength of stability capacity and production capacity were defined based on the grading evaluation results of four newly established stress indices, in which level 1 represented extremely low, level 2 represented low, level 3 represented medium, level 4 represented high, and level 5 represented extremely high. Based on the above results, 206 barley germplasms are divided into two classes by cluster analysis, with class 1 being high drought-adapted genotypes and class 2 being low drought-adapted genotypes (**Figure 10**).

In addition, the types of drought adaptability can be further subdivided according to SI and II levels. In class 1, counting from genotype 150 in a counterclockwise direction to genotype 206 belongs to type C (performing well only in stress environments); counting from genotype 190 in a counterclockwise direction to genotype 54 belongs to type A (performing well in both non-stress and stress environments). Similarly, in class 2, counting from genotype 8 in a counterclockwise direction to genotype 119 belongs to type B (performing well only in non-stress environments); counting from genotype 51 in a counterclockwise direction to genotype 66 belongs to type D (performing poorly in both non-stress and stress environments).

Furthermore, the stability differences of various drought-adapted types can be determined according to PI and EI. In type C, both PI and EI are greater than or equal to 3, with EI higher than PI, indicating that the tested genotypes exhibit good production potential and elasticity, and elasticity contributes more to high dry weight. In type A, PI ranges from 3 to 5, and EI ranges from 2 to 4, indicating that the tested genotypes have well production potential and different elastic performance. For example, the SI, II, PI, and EI levels of genotype 190 are 5, 5, 4, and 2, respectively, indicating that the high drought adaptation is mainly due to high potential, while the SI, II, PI, and EI levels of genotype 195 ranges 5, 4, 5 and 4, respectively, indicating that the high drought adaptation is the result of the combined action of high potential and high elasticity. In type B, PI is from 1 to 3, and EI is from 1 to 2, indicating poor performance under stress due to their low potential and elasticity. In type D, PI ranges from 1 to 3, and EI ranges from 2 to 5, indicating that the tested genotypes have poor production potential and different elastic performance. For example, the SI, II, PI, and EI levels of genotype 51 are 1, 2, 1, and 2, respectively, indicating that the low drought adaptation is the result of the combined action of low potential and elasticity, while the SI, II, PI, and EI levels of genotype 205 are 2, 1, 1, and 4, respectively, indicating that the low drought adaptation is mainly due to low potential.

In breeding practice, breeders tend to choose type A (performing well under both control and stress conditions). If production capacity is taken as the primary measure, the genotypes with SI, II, PI, and EI levels of 5, 5, 5, and 3, respectively, perform best, and their corresponding genotypes are 133, 129, 120, 74, 18, and 69. If the stability ability is taken as the primary measure, the genotypes with SI, II, PI, and EI levels of 5, 4, 5, and 4, respectively, performed best, and their corresponding genotypes are 195, 136, 128, 40, and 80.

## 4 Discussion

### 4.1 Dry weight serves as a reliable trait for assessing the growth of barley seedlings

Plant drought response and adaptation are complex processes, including morphological, physiological, and biochemical changes (Groen et al., 2022; Gupta et al., 2020). The uniform and strong seedlings are the most common and crucial criteria in early growth stage, although traits relative to regulation of photosynthesis, activation of antioxidant enzymes, and balance of osmotic regulation system have been used to evaluate the drought adaptability germplasms in recent years (Alharby et al., 2019; Wang et al., 2022). The total biomass of seedlings per unit sowing amount is the comprehensive embodiment of uniform and strong seedlings, and the detection of this trait has both validity and economic adaptability in the identification of high throughput germplasm resources. Seedling biomass including the parameters of dry weight and fresh weight, and the former more accurately reflects plant biomass accumulation compared to the latter (Huang et al., 2017; Younginger et al., 2017). Therefore, dry weight was used as the main observation criterion for assessing the growth of barley seedlings in the present study. The results indicated a varying degree of decrease in dry weight for 206 genotypes under 20% polyethylene glycol stress, consistent with the findings of Cai et al. (2020) (**Figure 1**). Moreover, previous studies often utilized limited genotypes or genetic populations for screening stress adaptation materials, potentially limiting the generalizability and accuracy of the results (Elakhdar et al., 2022). To address this, the current study tested 206 barley germplasms to assess the effects of drought stress on the quality of seedling dry weight. The results indicated a high CV in the dry weight of 206 germplasms (31.54%), which was even higher under drought conditions (61.14%) (**Figure 1**). This trend was different from the previous study (Cai et al., 2020), which means a higher diversity in the tested germplasms, and suitable for subsequent stress indices analysis.

### 4.2 Traditional stress indices have limitations in dividing drought adaptability of barley seedlings

In addition to diverse genetic and reliable traits, an accurate typing method is also crucial for identifying drought-adapted materials. Currently, stress indices like DC, GMP, MP, SSI, STI, and TOL are commonly applied to assess crop yield at the mature stage but are seldom utilized for evaluating seedling biomass (Ayed et al., 2021; Bao et al., 2023). However, the seedling stage is more suitable for the screening of drought-adapted varieties compared with the latter growth stages because seedlings are not affected by complex development and reproduction like fully developed plants, and their growth is more accessible to evaluate and prove (Peršić et al., 2022). Moreover, the selection of drought-adapted germplasms based on seedlings is also of great significance for water absorption and seedling establishment under limited water supply or extreme weather conditions (Sallam et al., 2019). Therefore, we introduced the above stress indices into the drought adaptability evaluation system of barley seedlings. The results showed that the CV of DC, GMP, MP, SSI, STI, and TOL were all higher than 30%, which well reflected the diversity of the tested germplasms (**Figure 3**). Among them, DC, GMP, MP, and STI exhibited a positive correlation with biomass, whereas SSI and TOL exhibited a negative correlation (**Figure 2**). These results are similar to those from previous study (Fernandez, 1992). The above indices reflected the diverse responses of genotypes to environmental changes. The genotypes with high drought adaptability selected by the former stress indices were 28, 40, 133, 69, 195, 74, 129, 65, 120, and 136, while those of the latter were 150, 14, 22, 151, 148, and 183. Unfortunately, there was no overlap in the high drought-adapted genotypes screened by the two types of indices (**Figure 3**). This outcome highlights the inadequacy of a single stress index in providing a comprehensive assessment of the drought resistance of each genotype. Additionally, the two types of indices encounter challenges arising from different directions of action, dimensions, and orders of magnitude, which complicates achieving an intuitive and comprehensive evaluation. Consequently, the traditional stress indices have considerable limitations in categorizing the drought adaptability of barley seedlings.

### 4.3 Newly developed indices are an effective tool for evaluating the drought adaptability of seedlings

Normalization methods are widely used to deal with all kinds of complex data sets, such as high-throughput proteomics, single-cell sequencing, and internal reference gene verification (de Andrade et al., 2017; Jin et al., 2020; Lause et al., 2021). Due to the shortcomings of stress indices in assessing seedling drought adaptability, the min-max scale and the negative min-max scale methods were used to eliminate the differences in action direction, dimension, and order of magnitude among stress indices in this study (**Figure 4**). Furthermore, since there are four positive stress indices and two negative stress indices, the mean normalized values of the two types of indices were then calculated to obtain the comprehensive stress indices: PI (Potential index) and EI (Elasticity index), and the former highlights the stress production potential and the latter highlights the stress production elasticity (**Figures 5**, **6**).

Dimensionality reduction algorithms are widely used in feature reduction, such as principal component analysis, partial least square analysis, and factor analysis (Liu et al., 2022a; Tsuyuzaki et al., 2020). Due to the strong correlation among the six stress indices, principal component analysis was used, and the highly correlated variables were reduced to new variables in the present study (**Figure 7**). As a result, two principal components were extracted, and the *R^2^* for the linear regression between PC1 and dry weight under drought conditions, as well as between PC2 and dry weight under control conditions, both exceeded that for the linear regression between six stress indices and dry weight under drought or control conditions (**Figures 7**, **8**). Finally, PC1 was named SI (Stress production index), and PC2 was named II (Ideal production index) (**Figure 9**).

There are many methods to distinguish different sample classifications, such as grading evaluation, cluster analysis, and so on (Dodig et al., 2019; Zappia and Oshlack, 2018). To facilitate a more intuitive observation of the numerical distribution characteristics of the four newly established stress indices, a grading evaluation scale was proposed, with each 20% interval of the total range representing a gradient. The grades are defined as follows: level 1 (extremely low), level 2 (low), level 3 (medium), level 4 (high), and level 5 (extremely high). The results showed that the classification results integrated the distribution pattern of the six stress indices well. Furthermore, the cluster analysis of four newly developed stress indices categorized 206 genotypes into two types of drought adaptation, demonstrating a less refined differentiation effect compared to the four drought adaptation crop types proposed by Fernandez (1992). However, the results of cluster analysis combined with the results of SI and II grading evaluation can accurately classify the differences in production performance of the tested germplasms and make up for the above deficiencies. What is more, the stability differences of four drought-adapted genotypes, encompassing stress elasticity and potential, could be further discerned by integrating the results of PI and EI grading evaluations. Ultimately, the genotypes with high production and high stability performance were selected, which were 133, 129, 120, 74, 18, 69, 195, 136, 128, 40 and 80, respectively (**Figure 10**).

## 5 Conclusion

The diverse genetic resources, reliable traits, and accurate genotyping methods are crucial for screening drought-adapted genotypes. The study utilized 206 germplasm resources as experimental materials, dry weight as the observed trait, and two genotyping methods to assess drought adaptability. Among them, traditional indices such as DC, GMP, MP, SSI, STI, and TOL as evaluation methods revealed inherent shortcomings, including insufficient accuracy and significant variations. However, four newly developed stress indices (SI, II, PI, and EI) effectively addressed the above issues. Furthermore, on the basis of grading evaluation and cluster analysis of the four new stress indices, a comprehensive, intuitive, and suitable high-throughput method was established, which fully considered the production and stability characteristics of the tested germplasm resources under different environments. Ultimately, 11 high drought-adapted genotypes with high stability and production capacities were screened, including 133, 129, 120, 74, 18, 69, 195, 136, 128, 40, and 80.

## Data Availability

The original contributions presented in the study are included in the article/**Supplementary Material**, further inquiries can be directed to the corresponding author.
