# Supplementary material for: Evaluation of barley genotypes for drought adaptability: based on stress indices and comprehensive evaluation as criteria
Source: Front Plant Sci. 2024 Aug 26;15:1436872. doi: 10.3389/fpls.2024.1436872 (PMC11381406; doi:10.3389/fpls.2024.1436872)
Supplement: Supplementary file 1 [file Table1.docx]

Supplementary Material

**Supplementary Table 1.** Barley accessions used for evaluation of drought adaptability in this study.

| Accession number | Accession name | Scientific name | Accession number | Accession name | Scientific name |
| --- | --- | --- | --- | --- | --- |
| 1 | 2014C/57 | *Hordeum vulgare* L. | 104 | Sampson | *Hordeum vulgare* L. |
| 2 | 2014C/93 | *Hordeum vulgare* L. | 105 | Conrad | *Hordeum vulgare* L. |
| 3 | 2016C/8 | *Hordeum vulgare* L. | 106 | Harrington | *Hordeum vulgare* L. |
| 4 | 2016C/165 | *Hordeum vulgare* L. | 107 | Scarlett | *Hordeum vulgare* L. |
| 5 | P17-7 | *Hordeum vulgare* L. | 108 | Cdc Rinty | *Hordeum vulgare* L. |
| 6 | P16-20 | *Hordeum vulgare* L. | 109 | Cdc Fleet | *Hordeum vulgare* L. |
| 7 | P18-3 | *Hordeum vulgare* L. | 110 | Empress | *Hordeum vulgare* var. *coeleste Linnaeus* |
| 8 | P18-7 | *Hordeum vulgare* L. | 111 | Kendall | *Hordeum vulgare* L. |
| 9 | P14-11 | *Hordeum vulgare* L. | 112 | Merit | *Hordeum vulgare* L. |
| 10 | P16-10 | *Hordeum vulgare* L. | 113 | Xanadu | *Hordeum vulgare* L. |
| 11 | P18-4 | *Hordeum vulgare* L. | 114 | Golden Promise | *Hordeum vulgare* L. |
| 12 | P19-1 | *Hordeum vulgare* L. | 115 | Berenic | *Hordeum vulgare* L. |
| 13 | P19-2 | *Hordeum vulgare* L. | 116 | Laiseyi | *Hordeum vulgare* L. |
| 14 | P19-4 | *Hordeum vulgare* L. | 117 | Fake | *Hordeum vulgare* L. |
| 15 | P19-7 | *Hordeum vulgare* L. | 118 | Heiyinrui | *Hordeum vulgare* L. |
| 16 | P19-8 | *Hordeum vulgare* L. | 119 | Polish barley 28 | *Hordeum vulgare* L. |
| 17 | P19-9 | *Hordeum vulgare* L. | 120 | Polish barley 29 | *Hordeum vulgare* L. |
| 18 | P17-4 | *Hordeum vulgare* L. | 121 | Polish barley 31 | *Hordeum vulgare* L. |
| 19 | Yuan 81-306 | *Hordeum vulgare* L. | 122 | Polish barley 32 | *Hordeum vulgare* L. |

Continued Supplementary Table 1.

| 20 | Zheguang | *Hordeum vulgare* L. | 123 | Polish barley 33 | *Hordeum vulgare* L. |
| --- | --- | --- | --- | --- | --- |
| 21 | Krkcuj | *Hordeum vulgare* L. | 124 | Polish barley 34 | *Hordeum vulgare* L. |
| 22 | Nadji | *Hordeum vulgare* L. | 125 | Polish barley 35 | *Hordeum vulgare* L. |
| 23 | Plenum | *Hordeum vulgare* L. | 126 | Polish barley 37 | *Hordeum vulgare* L. |
| 24 | Kimberly | *Hordeum vulgare* L. | 127 | Xi 02-942 | *Hordeum vulgare* L. |
| 25 | Kepin No.2 | *Hordeum vulgare* L. | 128 | Xi 02-948 | *Hordeum vulgare* L. |
| 26 | Riso 1508 | *Hordeum vulgare* L. | 129 | Xi 02-1134 | *Hordeum vulgare* L. |
| 27 | I FFI-9 | *Hordeum vulgare* L. | 130 | Xi 02-1146 | *Hordeum vulgare* L. |
| 28 | Ideal V69-72 | *Hordeum vulgare* L. | 131 | Xi 02-1236 | *Hordeum vulgare* L. |
| 29 | Nutans-244 | *Hordeum vulgare* L. | 132 | ND 12567 | *Hordeum vulgare* L. |
| 30 | klagesX (8537-68) 2 | *Hordeum vulgare* L. | 133 | ND 13299 | *Hordeum vulgare* L. |
| 31 | Diamant | *Hordeum vulgare* L. | 134 | ND 14636 | *Hordeum vulgare* L. |
| 32 | Nad ja | *Hordeum vulgare* L. | 135 | ND 15486 | *Hordeum vulgare* L. |
| 33 | Hilde | *Hordeum vulgare* L. | 136 | ND 4994-16 | *Hordeum vulgare* L. |
| 34 | Korat | *Hordeum vulgare* L. | 137 | ND 11231-11 | *Hordeum vulgare* L. |
| 35 | Hana | *Hordeum vulgare* L. | 138 | 2B98-5754-C | *Hordeum vulgare* L. |
| 36 | Mata | *Hordeum vulgare* L. | 139 | 2B98-5754-D | *Hordeum vulgare* L. |
| 37 | Manapou | *Hordeum vulgare* L. | 140 | 2B99-2657 | *Hordeum vulgare* L. |
| 38 | Kym | *Hordeum vulgare* L. | 141 | 2B00-0089 | *Hordeum vulgare* L. |
| 39 | Ma 19-125 | *Hordeum vulgare* L. | 142 | 2B00-0140 | *Hordeum vulgare* L. |
| 40 | Ma 19-126 | *Hordeum vulgare* L. | 143 | 2B00-0414 | *Hordeum vulgare* L. |
| 41 | Ma 19-127 | *Hordeum vulgare* L. | 144 | 2B00-0794 | *Hordeum vulgare* L. |
| 42 | Ma 19-128 | *Hordeum vulgare* L. | 145 | 2B01-1884 | *Hordeum vulgare* L. |

Continued Supplementary Table 1.

| 43 | Ma 19-129 | *Hordeum vulgare* L. | 146 | 2B01-1986 | *Hordeum vulgare* L. |
| --- | --- | --- | --- | --- | --- |
| 44 | Ma 19-130 | *Hordeum vulgare* L. | 147 | 2B01-2010 | *Hordeum vulgare* L. |
| 45 | Ma 19-132 | *Hordeum vulgare* L. | 148 | 2B10-4062 | *Hordeum vulgare* L. |
| 46 | Ma 19-133 | *Hordeum vulgare* L. | 149 | 2B10-4301 | *Hordeum vulgare* L. |
| 47 | Ma 19-134 | *Hordeum vulgare* L. | 150 | 2B10-4465 | *Hordeum vulgare* L. |
| 48 | Ma 19-136 | *Hordeum vulgare* L. | 151 | 2B10-4511 | *Hordeum vulgare* L. |
| 49 | Ma 19-138 | *Hordeum vulgare* L. | 152 | Z034Q016R | *Hordeum vulgare* L. |
| 50 | Ma 19-140 | *Hordeum vulgare* L. | 153 | Z171U038V | *Hordeum vulgare* L. |
| 51 | Ma 19-142 | *Hordeum vulgare* L. | 154 | BARI 210 | *Hordeum vulgare* L. |
| 52 | Ma 19-144 | *Hordeum vulgare* L. | 155 | BARI 282 | *Hordeum vulgare* L. |
| 53 | Ma 19-145 | *Hordeum vulgare* L. | 156 | Haploid barley 18 | *Hordeum vulgare* L. |
| 54 | Ma 19-146 | *Hordeum vulgare* L. | 157 | Haploid barley 19 | *Hordeum vulgare* L. |
| 55 | Ma 19-149 | *Hordeum vulgare* L. | 158 | 2007C/18 | *Hordeum vulgare* L. |
| 56 | Ma 19-150 | *Hordeum vulgare* L. | 159 | 2010C/12 | *Hordeum vulgare* L. |
| 57 | Ma 19-152 | *Hordeum vulgare* L. | 160 | 2011C/46 | *Hordeum vulgare* L. |
| 58 | Ma 19-153 | *Hordeum vulgare* L. | 161 | 2013WJ/3 | *Hordeum vulgare* L. |
| 59 | Ma 19-154 | *Hordeum vulgare* L. | 162 | 2013WJ/8 | *Hordeum vulgare* L. |
| 60 | Ma 19-155 | *Hordeum vulgare* L. | 163 | 2015C/27 | *Hordeum vulgare* L. |
| 61 | Ma 19-157 | *Hordeum vulgare* L. | 164 | Xinnong 2016/3 | *Hordeum vulgare* L. |
| 62 | Ma 19-158 | *Hordeum vulgare* L. | 165 | 2014C/99 | *Hordeum vulgare* L. |
| 63 | Ma 19-160 | *Hordeum vulgare* L. | 166 | 2014C/75 | *Hordeum vulgare* L. |
| 64 | Ma 19-170 | *Hordeum vulgare* L. | 167 | 2014C/101 | *Hordeum vulgare* L. |
| 65 | Ma 19-182 | *Hordeum vulgare* L. | 168 | 9810 | *Hordeum vulgare* L. |

Continued Supplementary Table 1.

| 66 | Ma 19-199 | *Hordeum vulgare* L. | 169 | HF05-1-11 | *Hordeum vulgare* L. |
| --- | --- | --- | --- | --- | --- |
| 67 | Ma 19-203 | *Hordeum vulgare* L. | 170 | 16-11006 | *Hordeum vulgare* L. |
| 68 | Ma 19-208 | *Hordeum vulgare* L. | 171 | 16-11008 | *Hordeum vulgare* L. |
| 69 | Ma 19-211 | *Hordeum vulgare* L. | 172 | C-18 | *Hordeum vulgare* L. |
| 70 | Ma 19-219 | *Hordeum vulgare* L. | 173 | H41-1 | *Hordeum vulgare* L. |
| 71 | Ma 19-220 | *Hordeum vulgare* L. | 174 | Vada | *Hordeum vulgare* L. |
| 72 | Gankenpi 4216 | *Hordeum vulgare* L. | 175 | Hamai 1 | *Hordeum vulgare* L. |
| 73 | Gankenpi 21119 | *Hordeum vulgare* L. | 176 | Xinpi No.6 | *Hordeum vulgare* var. *coeleste Linnaeus* |
| 74 | 11P5-004 | *Hordeum vulgare* L. | 177 | 0349-1 | *Hordeum vulgare* var. *coeleste Linnaeus* |
| 75 | 12P5-051 | *Hordeum vulgare* L. | 178 | Xinke No.1 | *Hordeum vulgare* var. *coeleste Linnaeus* |
| 76 | Kenpimai 17 | *Hordeum vulgare* L. | 179 | Yunke No.4 | *Hordeum vulgare* var. *coeleste Linnaeus* |
| 77 | Heipi No.2 | *Hordeum vulgare* L. | 180 | Longke No.7 | *Hordeum vulgare* var. *coeleste Linnaeus* |
| 78 | Heipi No.3 | *Hordeum vulgare* L. | 181 | 13-6927 | *Hordeum vulgare* var. *coeleste Linnaeus* |
| 79 | 0520-23 | *Hordeum vulgare* L. | 182 | BQ 15048-1 | *Hordeum vulgare* L. |
| 80 | 0521-6 | *Hordeum vulgare* L. | 183 | 2018C/102 | *Hordeum vulgare* L. |
| 81 | Z06-278-9 | *Hordeum vulgare* L. | 184 | 2020C/106 | *Hordeum vulgare* L. |
| 82 | Xinpi No.1 | *Hordeum vulgare* L. | 185 | Ganpi No.8 | *Hordeum vulgare* L. |
| 83 | Xinpi No.2 | *Hordeum vulgare* L. | 186 | 2018C/95 | *Hordeum vulgare* L. |
| 84 | Xinyin D5 | *Hordeum vulgare* L. | 187 | 2018C/81 | *Hordeum vulgare* var. *coeleste Linnaeus* |
| 85 | Xinyin D7 | *Hordeum vulgare* L. | 188 | Kang 18705 | *Hordeum vulgare* var. *coeleste Linnaeus* |
| 86 | Xinyin D9 | *Hordeum vulgare* L. | 189 | Xinke No.2 | *Hordeum vulgare* var. *coeleste Linnaeus* |
| 87 | 1109M050M | *Hordeum vulgare* L. | 190 | 0628 | *Hordeum vulgare* var. *coeleste Linnaeus* |
| 88 | 1090M066M | *Hordeum vulgare* L. | 191 | 14-3492 | *Hordeum vulgare* var. *coeleste Linnaeus* |

Continued Supplementary Table 1.

| 89 | Xinyin D10 | *Hordeum vulgare* L. | 192 | 13-51721-7 | *Hordeum vulgare* var. *coeleste Linnaeus* |
| --- | --- | --- | --- | --- | --- |
| 90 | Ganken No.6 | *Hordeum vulgare* L. | 193 | Longke No.8 | *Hordeum vulgare* var. *coeleste Linnaeus* |
| 91 | Gankenpi 0520 | *Hordeum vulgare* L. | 194 | 14-831 | *Hordeum vulgare* var. *coeleste Linnaeus* |
| 92 | Gankenpi 0215 | *Hordeum vulgare* L. | 195 | 14-712 | *Hordeum vulgare* var. *coeleste Linnaeus* |
| 93 | Kenpi No.6 | *Hordeum vulgare* L. | 196 | QTB 13 | *Hordeum vulgare* var. *coeleste Linnaeus* |
| 94 | Kenpi No.7 | *Hordeum vulgare* L. | 197 | Yanshike No.1 | *Hordeum vulgare* var. *coeleste Linnaeus* |
| 95 | Ganpi No.1 | *Hordeum vulgare* L. | 198 | QTB 11 | *Hordeum vulgare* var. *coeleste Linnaeus* |
| 96 | Ganpi No.3 | *Hordeum vulgare* L. | 199 | QB 27 | *Hordeum vulgare* var. *coeleste Linnaeus* |
| 97 | Ganpi No.6 | *Hordeum vulgare* L. | 200 | QB 14 | *Hordeum vulgare* var. *coeleste Linnaeus* |
| 98 | Ganpi No.7 | *Hordeum vulgare* L. | 201 | 0400-17 | *Hordeum vulgare* var. *coeleste Linnaeus* |
| 99 | Long 18F6-1309 | *Hordeum vulgare* L. | 202 | QB 16 | *Hordeum vulgare* var. *coeleste Linnaeus* |
| 100 | Long 18F6-1770 | *Hordeum vulgare* L. | 203 | QTB 25 | *Hordeum vulgare* var. *coeleste Linnaeus* |
| 101 | Poland | *Hordeum vulgare* L. | 204 | Longkemai No.4 | *Hordeum vulgare* var. *coeleste Linnaeus* |
| 102 | Tradition | *Hordeum vulgare* L. | 205 | Longkemai No.5 | *Hordeum vulgare* var. *coeleste Linnaeus* |
| 103 | Alexis | *Hordeum vulgare* L. | 206 | Longqing No.1 | *Hordeum vulgare* var. *coeleste Linnaeus* |
